# Sick Sinus Syndrome Can Be Associated with Postural Tachycardia Syndrome and Inappropriate Sinus Tachycardia Syndrome

**DOI:** 10.19102/icrm.2021.120503

**Published:** 2021-05-15

**Authors:** Paul R. Harnish, Pinang Shastri, Blair P. Grubb

**Affiliations:** ^1^Division of Cardiovascular Medicine, Department of Medicine, University of Toledo, Toledo, OH, USA; ^2^Department of Medicine, University of Toledo, Toledo, OH, USA

**Keywords:** Inappropriate sinus tachycardia, postural orthostatic tachycardic syndrome, sinus node dysfunction

## Abstract

As a known phenomenon, crossover between sinus node dysfunction and common atrial tachyarrhythmias—most notably, atrial fibrillation and atrial flutter—in older individuals has previously been seen. Here, we present one of the first case series demonstrating a similar relationship between sinus node dysfunction and much rarer etiologies of tachyarrhythmia—that is, postural tachycardia syndrome and inappropriate sinus tachycardia. The exact pathological mechanisms behind these arrhythmias as well as the observation of concurrent nodal dysfunction are poorly understood. Here, we propose both potential mechanistic pathways as well as an initial treatment algorithm for sinus node dysfunction based upon the existing evidence.

## Introduction

Here, we examine a rare but known phenomenon in which patients with persistently tachycardic baseline heart rates due to previously diagnosed postural orthostatic tachycardia syndrome (POTS) or inappropriate sinus tachycardia (IST) demonstrate a transition into periods of either transient or sustained symptomatic bradycardia. This is similar in scope to the known crossover between sinus node dysfunction and more common atrial tachyarrhythmias—most notably, atrial fibrillation and atrial flutter—in older individuals.^1,2^ Given the rarity of POTS and IST in the general population, the frequency as well as the mechanisms of this crossover remain unknown. We detail four separate cases, three of which eventually underwent pacemaker implantation and one of which may require similar treatment in the future.

## Case presentations

### Case 1

A 20-year-old woman with a known history of POTS and episodes of syncope starting at the age of 13 years presented for evaluation. At the beginning of her disease course, she experienced palpitations, light-headedness, and dizziness, with positional changes. Her symptoms progressed into her teenage years with two to three episodes of syncope per month and recurrent emergency room and physician visits. She was seen by multiple providers and had been labeled as a psychiatric patient until she was ultimately referred to our electrophysiology (EP) clinic due to elevated heart rates associated with her episodes. She underwent tilt-table testing and experienced symptomatic tachycardia up to 143 bpm; variations in blood pressure (BP) from 119/87 to 103/54; and, ultimately, syncope during the study. After a thorough review of her symptoms, she was diagnosed with autonomic dysfunction and orthostatic intolerance consistent with postural tachycardia syndrome. She was treated with intravenous saline infusions as well as ivabradine after failing multiple other medical therapies, including bupropion, fludrocortisone, nebivolol, and droxidopa. After some initial success with this regimen, she was later admitted to the hospital with debilitating episodes of bradycardia at the age of 19 years. All rate-controlling medications were discontinued and an ambulatory monitor was placed. She was found to be in persistent sinus bradycardia with rates in the 30s and 40s, which did not change with repositioning. Due to intractable symptoms despite withdrawal of all rate-controlling agents and a period of monitoring, she ultimately required placement of a dual-chamber pacemaker, with significant improvements in symptoms observed with atrial pacing at a burden of 60% to 70%.

### Case 2

A very accomplished 53-year-old female professional musician presented as a patient to our clinic with a long history of debilitating POTS and episodes of syncope, which significantly interfered with her ability to work as well as quality of life. She had undergone extensive evaluation with positive tilt-table testing findings at two prior centers consisting of tachycardia and hypotension prior to referral to our clinic. She had been trialed on multiple medications, including bupropion, droxidopa, and saline infusions, and had shown initial success with medical management with long-term follow-up (see **Table 1** for the complete list). About 12 years from the time of her initial diagnosis, however, she experienced an abrupt change in the nature of her syncopal episodes with an advancement in frequency and severity of events and a loss of her usual initial prodrome, raising the potential for significant future trauma. An implantable loop recorder was placed, which revealed new episodes of profound sinus bradycardia with rates dipping into the range of 20 to 39 bpm. Following this discovery, a dual-chamber pacemaker was placed with near-complete resolution of symptoms and requiring pacing at a 60% to 70% burden. She has since returned to a near-normal quality of life a few years out from device placement.

### Case 3

A 49-year-old woman with a known history of Sjögren’s syndrome, mitochondrial myopathy, and hypermobility syndrome presented for assessment. Her disease course began with autonomic neuropathy, which included numbness and a sensation of tingling/pruritus in the arms and legs. She was initially diagnosed with autonomic dysfunction at an outside institution after developing episodes of positional tachycardia. She subsequently presented to our EP clinic after years of wide-ranging rates that were initially predominately tachycardic but which progressed into episodes of severe bradycardia. Despite attempts to use multiple medications, including pyridostigmine and dextroamphetamine, she progressed into frank chronotropic incompetence with an average heart rate of 40 bpm. Despite removal of prior rate-controlling agents and attempts at medical therapy, she ultimately required placement of a dual-chamber pacemaker, with subsequent significant improvements in her symptoms. During follow-up pacemaker interrogations, she has proven to require atrial pacing at an 80% burden with cessation of her prior tachycardic episodes.

### Case 4

A 32-year-old man with a history of small-fiber neuropathy and Sjögren’s syndrome initially presented with episodes of a racing heart accompanied by light-headedness. He underwent an initial workup, including tilt-table testing, which demonstrated a postural tachycardia response from an initial baseline rate of 60 bpm up to 144 bpm. His tilt test was actually halted early at 20 minutes and 70° of tilt due to a drop in both systolic and diastolic BPs down to 87/70 mmHg. He was initially diagnosed with autonomic dysfunction given the presence of comorbid disease and was followed for a period of time on ivabradine. He then experienced progression into episodes of extreme bradycardia with rates in the 30s as documented by his implantable loop recorder. Around the same time as the worsening of his rhythm-related symptoms, he was diagnosed with early-onset Parkinson’s disease. This serves as an example of another known phenomenon in which autonomic dysfunction precedes neuromuscular symptoms. He is currently being managed medically with cessation of rate-controlling medicines but may eventually require pacing if symptoms persist and prove to correlate with his loop tracings.

## Discussion

POTS is characterized as a form of dysautonomia resulting in symptoms of orthostatic intolerance such as light-headedness, exercise intolerance, near-syncope with positional changes, fatigue, sweating, tremor, anxiety, and palpitations the typically resolve upon lying down. For many patients, this leads to a profound drop in quality of life as they suffer a decline in functional status even when conducting everyday tasks such as ascending the stairs or getting out of bed. This extends across a wide range of ages, including the very young. The criteria for diagnosis consist of a resting heart rate of greater than 120 bpm on standing or an increase in heart rate by 30 bpm from baseline after standing for 10 minutes. BP is usually preserved upon position change as compared with in the context of orthostatic hypotension, which is the hallmark of autonomic failure. Exclusion of confounding conditions (dehydration, anemia, hypothyroidism) or medications such as vasodilators, diuretics, antidepressants, or anxiolytic agents must be established prior to diagnosis.^3^

Primary and secondary forms of POTS are classified based on the proposed etiology of symptoms. Primary partial dysautonomia (or neurogenic) POTS is thought to a pathologic immune-mediated response via serum auto-antibodies to α1-adrenergic receptors in vascular smooth muscle, muscarinic M4 receptors, and α3-Ach receptors on peripheral ganglia.^4^ Hyperadrenergic states, usually the result of a genetic predisposition, can lead to increased norepinephrine (NE) levels which, together with impaired clearance or decreased uptake, can manifest as anxiety, tremulousness, and elevated diastolic BP.^5^ Metabolic, systemic, and autoimmune diseases may also manifest as POTS. Common culprits include diabetes mellitus, amyloidosis, heavy metal poisoning, Sjögren’s syndrome, hypermobility syndrome, and paraneoplastic syndrome. In addition, it has been found to be inducible following successful slow pathway atrioventricular nodal ablation.^6^

Distinguishing POTS from similar syndromes depends on adherence to specific clinical criteria, with the differential including IST syndrome, chronic fatigue syndrome, pheochromocytoma, and neurocardiogenic syncope. Specifically, IST has overlapping features with POTS, which can make proper diagnosis a nebulous task as both disorders result in an increase in heart rate with minimal activity. However, POTS is usually associated with elevations in heart rate in response to changes in the body position, whereas this is possible but not essential to make a diagnosis of IST. Both conditions can be severely debilitating to an affected individual, with at least one reported case of an individual with IST experiencing tachycardia-induced cardiomyopathy.^7^

Treatment of these related conditions is usually directed toward the modification of autonomic responses as well as rate control initially through medical therapy followed by radiofrequency sinus node ablation for refractory cases. Hyperadrenergic forms of POTS may respond to agents such as labetalol and clonidine due to their sympatholytic activity.^8^ Low-dose propranolol has also demonstrated efficacy in reducing tachycardia and achieving symptomatic control in POTS.^9^ Perturbations in serotonin production and regulation can also affect BP and heart rate, signifying a role for selective serotonin reuptake inhibitors in combination with serotonin and NE reuptake inhibitors as a potential adjunct therapy regimen for POTS via the stimulation of the standing vasoconstriction reflex.^10^ Other medications that have demonstrated success in select cases include fludrocortisone, midodrine, methylphenidate, and pyridostigmine for orthostatic syndromes.^11–13^ A promising therapy that has been gaining traction recently is ivabradine, a funny sodium channel blocker (iF blocker) that has demonstrated effects including reducing the heart rate and providing symptomatic relief from POTS as well as IST without significant concomitant drops in BP.^7,14,15^ More invasive methods such as pacemaker placement have also demonstrated efficacy in orthostatic intolerance syndromes that are refractory to medical treatment, specifically with the use of devices capable of responding to BP variations.^16^

Clear mechanisms have yet to be elucidated but include impaired vascular innervation, elevated NE concentrations, α- and β-receptor sensitivity, and baroreceptor dysfunction.^17^ Particularly, venous dysfunction secondary to impaired innervation can lead to pooling of blood in the legs and improper redistribution of blood to the peripheral circulation, leading to an increase in sympathetic receptor activity on the heart without a concurrent increase in vasoconstriction. Over time, this mismatch in autonomics and circulation may result in circulatory collapse.^8^ Similar mechanisms behind POTS and IST have been proposed, including intrinsically high sinus node automaticity rates due to ion channel dysfunction and alterations in autonomic nervous system tone together with decreased cardiovagal tone. In particular, abnormal autonomic modulation with elevated sympathetic and diminished parasympathetic tone has been shown to be a component of both POTS and IST.^18^ In addition, in vitro studies have reported the presence of circulating anti–β-adrenergic receptor antibodies leading to persistent, incremental production of cyclic adenosine monophosphate in IST, suggesting a role for autoimmunity in the development of the condition.^19,20^ A deficiency in NE transportation due to a single point mutation has also been identified, which can lead to a lack of clearance of NE, triggering excessive sympathetic activation.^21^

Given the aforementioned mechanisms for POTS and IST, many previously proposed mechanisms for the overlap between much more common tachycardias—most notably, atrial fibrillation—and sinus node dysfunction may serve as potential avenues to explain the observed transition from pathological tachycardia into bradycardia in these in these rarer cases. The first potential mechanism is that increased intrinsic automaticity due to a genetic channelopathy may induce a more rapid rate of nodal fibrosis. As previously suspected for other types of predominately tachycardic dysrhythmias such as atrial fibrillation, there is likely a degree of atrial anatomical and electrophysiological remodeling that occurs with persistent inappropriately elevated rates. While the etiologies in these cases—namely, POTS and IST—for the elevated rate are much rarer phenomena, the mechanism by which the sinus node degenerates may very well be similar. We know that radiofrequency-induced inflammation and fibrosis of the sinoatrial node lead to dysfunction, with progression into bradycardia. If the channelopathy confines itself specifically to the pacemaker cells of the sinus node, it is reasonable to suppose that they may be prone to a faster rate of fibrosis or dysfunction. Additionally, a second possible mechanism is that, if the pathological driving force behind the initially inappropriately elevated heart rate is for one reason or another halted, the long-term adaptation already undertaken by the central nervous system to combat this process in the form of increased baseline vagal tone would now stand unopposed. This may be the case, for example, in those patients with autoimmune-mediated disease with cessation of auto-antibody production or some new form of antagonism to the antibodies.

Finally, the third and, likely, the hardest to target is a shift in intrinsic autonomic tone in those patients with significant underlying dysautonomia. In the simplest terms, this would constitute a new shift consisting either of significant downregulation of a baseline high sympathetic tone or upregulation of a baseline low parasympathetic tone, resulting in a transition from tachycardia to bradycardia. With at least some proposed theories available to explain the transition point, a rational scheme for treatment and prevention can be postulated. In the case of progressive nodal fibrosis, if the rate of nodal degradation is linked to the degree of tachycardia, then appropriate rate control is paramount. This would include traditional agents such as β-blockers and central calcium channel blockers as well as newer agents, including iF channel blockers. In the case of autoimmune antibody formation, it is reasonable to presume that, over time, these patients develop increased baseline vagal tone. If the offending antibody is antagonized or its production halted, a transition into initial bradycardia is to be expected. This group would stand to benefit from targeted antibody receptor antagonism or immune suppression to reduce the production of the offending antibody. Following the identification and treatment of the driving pathologic force as well as discontinuation of any other medications that may worsen bradycardia, careful observation may prove to be an effective strategy. Lastly, if severe deviation in sympathetic or parasympathetic tone due to dysautonomia is present, there remain few additional therapies outside of mitigating downstream effects. As a last option, patients with progression from POTS or IST into symptomatic bradycardia may ultimately require pacing support due to unacceptably severe limitations in the quality of life.

**Figure 1:**
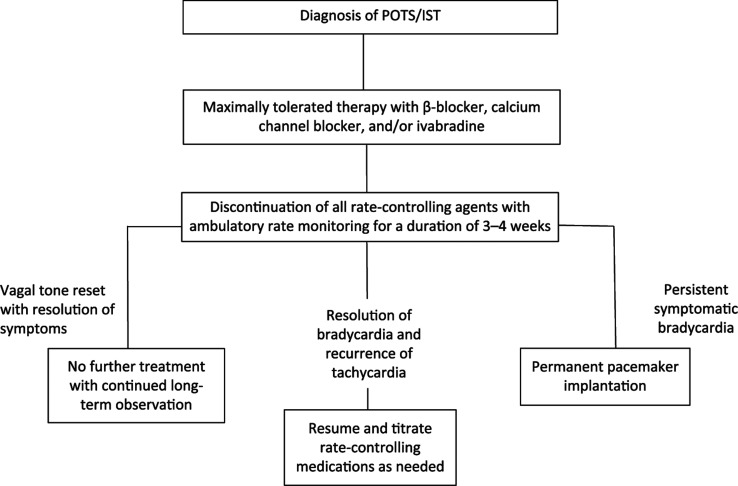
Proposed treatment pathway. CCB: calcium channel blocker.

This framework allows for the proposal of an initial treatment strategy for patients with an appropriate diagnosis of IST or POTS **(Figure 1)**. Maximally tolerated doses of a β-blocker or calcium channel blocker as well as an iF channel blocker should be considered as primary therapy to limit any associated nodal fibrosis. For patients who demonstrate a transition in baseline rate and symptoms from tachycardia to maintained bradycardia, an event monitor should be deployed and all offending rate-controlling agents should be halted. If bradycardic rates are persistently and inappropriately maintained with associated symptoms, a careful observation period of at least three to four weeks may allow for readjustment in baseline vagal tone. However, if symptoms continue despite these initial steps, the patient should be considered as a candidate for long-term pacing.

## Conclusion

As a rare but recurrent phenomenon, it has been observed in our clinic that patients suffering from POTS as well as IST have an increased propensity for progression into sinus node dysfunction consisting of episodes of either transient or sustained symptomatic bradycardia. A proven mechanism for this transition as well as the best management strategies for these cases remain unknown. We suspect the mechanisms of degeneration of the sinus node may be similar to those seen with other tachycardic dysrhythmias, such as atrial fibrillation or atrial flutter. With this case series, we hope to introduce the observation into the literature as a seed for ongoing investigation and to propose our own preliminary management algorithm.

### Limitations

This paper stands as an attempt to describe a witnessed phenomenon at a specialized syncope clinic. Due to the inherently small populations of these patients, the crossover between sinus node dysfunction and POTS/IST is suggested but unproven. A number of patients were evaluated at other centers before being referred to us and every effort was made to accurately summarize prior testing; however, the record may have missing components. Of note, it is a significant limitation that diagnostic electrophysiology studies were not performed preceding pacemaker implantation in these patients.

## Figures and Tables

**Table 1:** Patient Characteristics

Case	Associated Symptoms	Medications/Therapies Attempted	Tilt-table Testing	Comorbid Conditions	Pacemaker Implantation (Mode)
1	• Atypical chest pain • Recurrent syncopal episodes • Palpitations • Nausea • Diaphoresis	• High-salt diet • Florinef (D) • Ivabradine • Modafinil • Midodrine (D) • Droxidopa (D) • Anticholinergics (D) • Bupropion • Dofetilide • Intravenous hydration therapy	Tachycardia increased up to 143 bpm and BP varied from 119/87 to 103/54; patient experienced dizziness and an episode of syncope during the study	• Anxiety • Depression • Obesity	Yes (DDD)
2	• Syncopal episodes • Exercise intolerance • Palpitations • Light-headedness • Dizziness	• Bupropion • Droxidopa (D) • Midodrine (D) • Citalopram (D) • Pyridostigmine (D) • Modafinil • Intravenous hydration therapy	Tilt-table testing was performed at two prior centers before referral to our clinic, with findings consisting of tachycardia and hypotension with presyncopal symptoms	• Hypermobility syndrome	Yes (DDD)
3	• Autonomic neuropathy • Exercise intolerance • Syncope • Frequent headaches • Chronic fatigue	• Pyridostigmine • Dextroamphetamine • Topiramate (D)	Not performed	• Sjögren’s syndrome • Mitochondrial myopathy	Yes (DDD)
4	• Light-headedness • Presyncope • Palpitations	• Pyridostigmine • Tadalafil • Midodrine • Modafinil • Ivabradine • Bupropion • Dextroamphetamine (D)	Tachycardia increased up to 144 bpm and BP dropped to 87/70 with presyncopal symptoms	• Sjögren’s syndrome • Small-fiber neuropathy	No

BP: blood pressure; D: discontinued.
